# Supplementary material for: A high-resolution mRNA expression time course of embryonic development in zebrafish
Source: eLife. 2017 Nov 16;6:e30860. doi: 10.7554/eLife.30860 (PMC5690287; doi:10.7554/eLife.30860)
Supplement: Supplementary file 6. [file elife-30860-supp6.zip › biolayout-clusters-files/Cluster159-genes.html]

Cluster159


# Cluster159: Genes

| Ensembl ID | Gene Name | Chr | Start | End | Biotype |
| --- | --- | --- | --- | --- | --- |
| ENSDARG00000012763 | arl13b | 1 | 32935662 | 32962227 | protein\_coding |
| ENSDARG00000053656 | lypla2 | 19 | 44288547 | 44306974 | protein\_coding |
| ENSDARG00000039649 | mtif3 | 5 | 67293916 | 67301032 | protein\_coding |
| ENSDARG00000056600 | papss2b | 12 | 17337075 | 17357204 | protein\_coding |
| ENSDARG00000012248 | rgma | 18 | 24933082 | 24945528 | protein\_coding |
| ENSDARG00000026845 | rhoaa | 8 | 14132862 | 14142098 | protein\_coding |
| ENSDARG00000079687 | rnf10 | 8 | 41252761 | 41266135 | protein\_coding |
| ENSDARG00000057287 | slc25a16 | 13 | 18048104 | 18064950 | protein\_coding |
| ENSDARG00000017014 | ywhae2 | 7 | 71766327 | 71783613 | protein\_coding |
